# Causal relationship between sleep characteristics and thyroid function: A bidirectional Mendelian randomization study

**DOI:** 10.1097/MD.0000000000040516

**Published:** 2024-11-15

**Authors:** Zonghang Jia, Zhonghui Li, Yujie Li

**Affiliations:** a The First Clinical College of Shandong University of Traditional Chinese Medicine, Jinan, Shandong, China; b Department of Geriatrics, The Second Affiliated Hospital of Shandong University of Traditional Chinese Medicine, Jinan, Shandong, China.

**Keywords:** causal correlation, hyperthyroidism, hypothyroidism, Mendelian randomization, sleep characteristics

## Abstract

**Background:**

Previous researches have revealed some links between thyroid function and sleep characteristics, however it remains unclear which one causes the other. The purpose of this study was to investigate the potential causal relationship between hyperthyroidism, hypothyroidism, and sleep characteristics.

**Methods:**

We utilized aggregated data from published genome-wide association studies (GWAS) to select genetic instruments for sleep variables. The 5 sleep-related traits (chronotype, short sleep duration, long sleep duration, daytime sleepiness, and insomnia) were associated with distinct genetic variants chosen as instrumental factors. Employing MR Egger’s analysis of Mendelian randomization (MR), weighted median, weighted mode, and inverse variance weighted (IVW) methods to assess the 5 sleep traits in relation to hyperthyroidism and hypothyroidism, we subsequently conducted inverse MR analysis to examine the causal relationship between thyroid function and the 5 sleep characteristics.

**Results:**

The IVW technique did not reveal a causal association between chronotype, short sleep duration, long sleep duration, daytime sleepiness, or insomnia and the risk of abnormal thyroid function in the study investigating the influence of sleep characteristics on this risk. The outcomes of the IVW approach were consistent with the remaining 3 methods. The IVW, weighted median, MR Egger, and weighted mode methods in the reverse magnetic resonance imaging investigation did not yield evidence of a causative association between the risk of time type, long sleep duration, and insomnia and abnormal thyroid function. In contrast, the weighted median and weighted mode methods showed a possible causal relationship between hypothyroidism and short sleep duration and daytime sleepiness. Sensitivity analyses showed that the results were robust and no pleiotropy or heterogeneity was detected.

**Conclusion:**

More precisely, our analysis did not uncover any indication of a reciprocal causal link between thyroid function and genetically predicted sleep characteristics.

## 1. Introduction

As the largest endocrine gland in the human body, the thyroid secretes hormones that are essential for growth, development, and metabolism.^[1]^ Hyperthyroidism and hypothyroidism are the 2 primary abnormalities of thyroid function. According to research, the prevalence of hyperthyroidism is 1.5%, while the prevalence of hypothyroidism is 5%, with both rates currently on the rise.^[2]^ Various systemic illnesses, including cardiovascular, gastrointestinal, and neurological conditions, have been associated with hyperthyroidism and hypothyroidism. Sleep, a vital physiological restorative process crucial to human health, plays a role in maintaining and influencing every physiological function of the body.^[3–5]^ Each year, millions of Americans experience sleep-related disorders, and disruptions or deprivation of sleep can have significant and far-reaching effects on health.^[6,7]^ According to a survey, just half of Americans can keep up a regular sleep schedule,^[8]^ and sleep disorders are a problem in other nations as well.^[9]^ Numerous investigations have reported that diurnal changes impact thyroid hormone secretion,^[10,11]^ and free triiodothyronine (FT3) levels have been demonstrated to be negatively correlated with sleep length.^[12]^

Mendelian randomization (MR) technique is a statistical method used to study causal relationships between variables in observational studies. Compared with standard observational studies, MR has significant advantages in improving the reliability of findings by effectively reversing causal relationships and reducing the influence of confounding factors.^[13]^ Thyroid function and a number of disorders, including the metabolic syndrome,^[14]^ Parkinson’s disease,^[15]^ and intestinal flora,^[16]^ have been studied causally using MR analysis. The purpose of the present study was to evaluate the bidirectional causal association between sleep characteristics and hyperthyroidism and hypothyroidism, since the relationship between sleep characteristics and thyroid function is currently unknown. This study will contribute to the causal relationship between thyroid function and sleep, which may aid in prevention and treatment.

## 2. Materials and methods

### 2.1. Research methods

**Figure 1.**
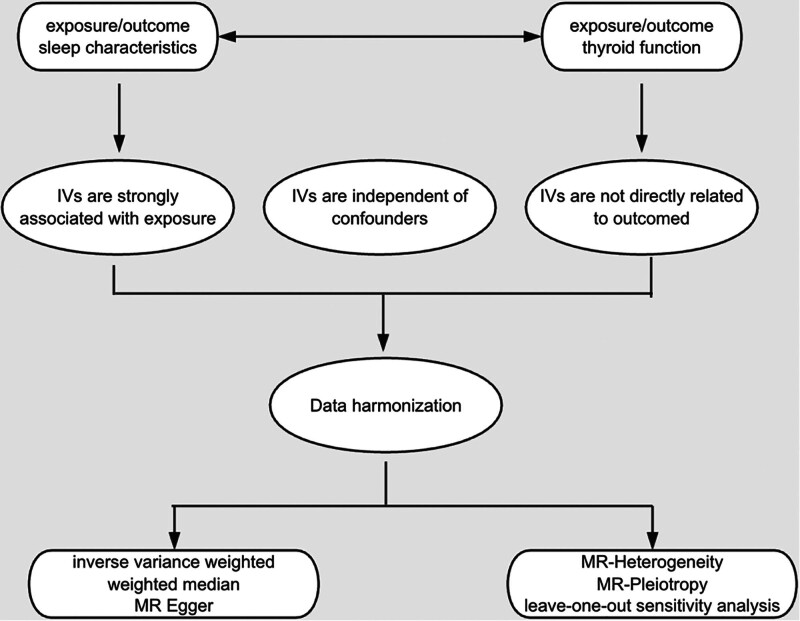
Workflow of Mendelian randomization study. IV = instrumental variables, MR = Mendelian randomization.

The design methodology for this study is shown in Figure 1. In order to ascertain the causal association between exposures and outcomes, we employed MR analysis in this work, which circumvents the bias issue in other research methodologies by looking for exposures using genetic variants.^[17,18]^ In order to evaluate the bidirectional causation between sleep characteristics and thyroid function, our study employed a 2-sample MR analysis. No new ethical permissions were required for this study as the data were primarily reanalyzed from previously published data obtained from public sources. Furthermore, our findings were reported in accordance with the MR-STROBE criteria.^[19]^

### 2.2. Data sources

Data on sleep characteristics were obtained from the UK Biobank Sleep Traits GWAS:Self-report (insomnia associations), which are publicly available (http://www.kp4cd.org/softet_downloads/sleep). Data on time type, which is a person’s preference for sleep characteristics, were obtained from the UK Biobank of 403,195 individuals of European ancestry;^[20]^ data on long and short sleep duration were obtained from the UK Biobank of 446, 118 adults of European ancestry in GWAS associations. Sleep duration of <7 hours was defined as short sleep (N = 106,192), and sleep duration of > 9 hours was defined as long sleep (N = 34,184);^[21]^ data on daytime sleepiness were from GWAS analyses of 452,071 individuals from the UK Biobank;^[22]^ data on insomnia were from the UK Biobank (N = 386,533) and from the 23 and Me (N = 944, 477) of a total of 1331,010 individuals with genetic associations;^[23]^ data for thyroid function were all from the FinnGen consortium version R9.^[24]^ Data on hyperthyroidism came from 367,578 individuals (5590 cases and 361,988 controls); data on hypothyroidism came from 314,995 individuals (40,926 cases and 274,069 controls), and participants were predominantly of European ancestry in our data sources for the MR analysis (Table 1).

**Table 1:** Details of the GWAS summary-level data.

Traits	N	Consortium	Data accession address
Time type	403,195	UKB	http://www.kp4cd.org/softet_downloads/sleep
Long sleep duration	34,184	UKB	http://www.kp4cd.org/softet_downloads/sleep
Short sleep duration	106,192	UKB	http://www.kp4cd.org/softet_downloads/sleep
Daytime sleepiness	452,071	UKB	http://www.kp4cd.org/softet_downloads/sleep
Insomnia	1331,010	23and Me and UKB	http://www.kp4cd.org/softet_downloads/sleep
Hyperthyroidism	367,578	Finngen	https://r9.finngen.fi/
Hypothyroidism	314,995	Finngen	https://r9.finngen.fi/

GWAS = genome-wide association studies, UKB = UK Biobank.

### 2.3. Selection of instrumental variables

The criteria for genetic variants to be instrumental variables (IVs) in this study were as follows^[25]^: the IVs needed to be strongly associated with sleep traits; the IVs needed to be independent of confounders related to sleep traits and thyroid function; besides, the IVs were associated only with sleep trait outcomes and had no direct correlation with thyroid function, and they could only have an effect on thyroid function through sleep traits. First we extracted SNPs that were strongly associated with sleep features from published data, where a looser threshold (*P* < 1E−5) was used for certain sleep features in order to include more relevant SNPs.^[26]^ To ensure that the instruments used for exposure were independent, we excluded SNPs that were in linkage disequilibrium (*r*^2^ < 0.001, kb = 10,000)^[27]^ and further excluded through the PhenoScanncer database (http://www.phenoscanner.medschl.cam.ac.uk/) confounding factors. We then extracted instrumental variables for the above sleep characteristics in the hyperthyroidism and hypothyroidism GWAS. Finally we reconciled the exposure data with the outcome data, meaning that the effects of SNPs on exposure and on outcome each corresponded to the same allele to exclude palindromic SNPs. We also computed the *F* statistic to eliminate bias due to weak instrumental variables in the results. We considered SNPs to be strong instrumental variables with small weak instrumental bias when the statistic *F* > 10^[26]^ was calculated for all relevant IVs in this MR study, indicating that they are less susceptible to weak IV bias.

### 2.4. Data analysis

The main methods utilized in this study included IVW, weighted median, weighted mode, and MR-Egger’s MR technique. Among these, the primary research method is IVW, which is an extension of the Wald ratio estimator based on the concept of meta-analysis.^[28]^ The weighted mean and weighted median approaches necessitate that at least 50% of the SNPs meet the criterion of being valid instrumental variables, while the other 3 methods served as supplementary approaches.^[29]^ The weighted median method has a lower power to test for causal effects, but also less bias.^[30]^ In contrast to IVW, the MR-Egger method takes into account the presence of an intercept term, and when horizontal pleiotropy is present, the MR-Egger method also provides an assessment of bias.^[31]^

For the sensitivity analysis in this study, the Cochran’s *Q* test was first used for detecting heterogeneity; Cochran’s *Q* test is mainly used to explore heterogeneity due to multiplicity or other reasons.^[32]^ The MR-Egger test for horizontal pleiotropy was subsequently employed. Furthermore, sensitivity analyses were conducted using the leave-one-out method, where each SNP was excluded 1 at a time, and the remaining SNPs were reanalyzed as instrumental variables for IVW effects. This approach aimed to evaluate the impact of individual SNPs on the analysis results and assess the stability of the study findings. Finally, analyses were performed by the software package TwoSample MR in R (version 4.3.1).

## 3. Results

### 3.1. Time type and thyroid function

The MR results in this section are based on IVs screened at the genome-wide significance threshold (*P* < 5e^−8^). Upon assessment, the causal effect of time type on thyroid function based on 11 IVs after performing a linkage disequilibrium screen (*r*^2^ < 0.001, kb = 10,000), and exclusion of outcome-associated SNPs. The IVW results showed that time type was not associated with the risk of hyperthyroidism (OR = 0.99, 95% CI: 0.95–1.04, *P* = .81), and hypothyroidism (OR = 1.34, 95% CI: 0.96–1.87, *P* = .09; Tables 2 and 3). The other 3 methods were consistent with the IVW results. In the reverse MR study, IVW results showed that hyperthyroidism (OR = 0.99, 95% CI: 0.95–1.04, *P* = .81), and hypothyroidism (OR = 0.98, 95% CI: 0.95–1.00, *P* = .09) were not associated with the risk of temporal type (Tables 4 and 5), and the other 3 methods were consistent with the IVW results. In addition, the results of the MR-Egger test for horizontal polytropy indicated that the MR analysis was not affected by any potential effect of horizontal polytropy (*P* > .05; Tables 6–9). Ultimately, the sensitivity analysis using the leave-one-out method confirmed the robustness of the MR results, as no key SNPs were found to have a significant impact on the results upon exclusion.

**Table 2:** Results of the causal effect of sleep characteristics on hyperthyroidism.

Exposure	N SNPs	Method	OR (95% CI)	*P*
Time type	11	IVW	0.71 (0.42, 1.19)	.19
		Weighted median	0.94 (0.46, 1.90)	.86
		MR-Egger	0.45 (0.07, 2.77)	.41
		Weighted mode	1.07 (0.38, 3.02)	.90
Daytime sleepiness	10	IVW	0.61 (0.05, 6.89)	.69
		Weighted median	0.20 (0.01, 3.46)	.27
		MR-Egger	0.03 (0.01, 30.99)	.35
		Weighted mode	0.17 (0.01, 9.25)	.41
Long sleep duration	8	IVW	0.55 (8.59e^−03^, 35.56)	.78
		Weighted median	0.27 (9.33e^−04^, 75.83)	.65
		MR-Egger	0.02 (5.58e^−07^, 1088.20)	.52
		Weighted mode	0.45 (4.99e^−05^, 4142.30)	.87
Short sleep duration	5	IVW	0.30 (6.15e^−03^, 1.46e^+01^)	.54
		Weighted median	0.50 (4.49e^−03^, 5.55e^+01^)	.77
		MR-Egger	4706.03 (9.34e^−05^, 2.37e^+11^)	.42
		Weighted mode	0.60 (1.86e^−03^, 1.95e^+02^)	.87
Insomnia	9	IVW	0.53 (1.00e^−01^, 2.80)	.46
		Weighted median	0.79 (8.70e^−02^, 7.13)	.83
		MR-Egger	1.32 (1.97e^−05^, 88919.25)	.96
		Weighted mode	1.98 (6.49e^−02^, 60.27)	.71

IVW = inverse variance weighted.

**Table 3:** Results of the causal effect of sleep characteristics on hypothyroidism.

Exposure	N SNPs	Method	OR (95% CI)	*P*
Time type	13	IVW	1.34 (0.96, 1.87)	.09
		Weighted median	1.75 (1.26, 2.41)	.01
		MR-Egger	4.08 (1.51, 10.98)	.02
		Weighted mode	1.79 (1.14, 2.81)	.03
Daytime sleepiness	10	IVW	1.46 (0.37, 5.80)	.59
		Weighted median	0.90 (0.26, 3.11)	.87
		MR-Egger	0.36 (0.01, 20.87)	.63
		Weighted mode	0.79 (0.20, 3.05)	.74
Long sleep duration	10	IVW	2.11 (0.41, 11.02)	.37
		Weighted median	1.43 (0.17, 11.68)	.74
		MR-Egger	0.03 (0.01, 1.84)	.13
		Weighted mode	1.13 (0.04, 33.94)	.94
Short sleep duration	6	IVW	1.29 (0.35, 4.80)	.71
		Weighted median	1.31 (0.24,7.07)	.75
		MR-Egger	111.23 (0.20, 63152.42)	.22
		Weighted mode	1.74 (0.16, 18.71)	.67
Insomnia	10	IVW	0.92 (0.47, 1.79)	.80
		Weighted median	0.94 (0.40, 2.19)	.88
		MR-Egger	0.41 (0.02, 8.15)	.58
		Weighted mode	0.97 (0.26, 3.63)	.96

IVW = inverse variance weighted.

**Table 4:** Results of the causal effect of hyperthyroidism on sleep characteristics.

Outcomes	N SNPs	Method	OR (95% CI)	*P*
Time type	5	IVW	0.99 (0.95, 1.04)	.81
		Weighted median	0.98 (0.94, 1.01)	.21
		MR-Egger	1.02 (0.87, 1.19)	.84
		Weighted mode	0.97 (0.94, 1.01)	.27
Daytime sleepiness	5	IVW	1.01 (0.99, 1.02)	.49
		Weighted median	1.01 (1.00, 1.03)	.11
		MR-Egger	1.02 (0.97, 1.06)	.53
		Weighted mode	1.01 (1.00, 1.03)	.19
Long sleep duration	5	IVW	1.00 (0.99, 1.01)	.39
		Weighted median	1.00 (0.99, 1.01)	.49
		MR-Egger	1.00 (0.98, 1.02)	.90
		Weighted mode	1.00 (0.98, 1.01)	.57
Short sleep duration	5	IVW	0.99 (0.98, 1.00)	.19
		Weighted median	0.99 (0.98, 1.01)	.22
		MR-Egger	0.97 (0.94, 1.00)	.45
		Weighted mode	0.99 (0.97, 1.00)	.21
Insomnia	5	IVW	1.01 (0.99, 1.02)	.33
		Weighted median	1.01 (0.99, 1.03)	.36
		MR-Egger	0.99 (0.95, 1.03)	.53
		Weighted mode	1.01 (0.99, 1.03)	.37

IVW = inverse variance weighted.

**Table 5:** Results of the causal effect of hypothyroidism on sleep characteristics.

Outcomes	N SNPs	Method	OR (95% CI)	*P*
Time type	10	IVW	0.98 (0.95, 1.00)	.09
		Weighted median	0.98 (0.96, 1.01)	.15
		MR-Egger	0.98 (0.93, 1.03)	.39
		Weighted mode	0.98 (0.95, 1.01)	.24
Daytime sleepiness	10	IVW	0.99 (0.98, 1.00)	.11
		Weighted median	0.99 (0.98, 1.00)	.01
		MR-Egger	0.99 (0.97, 1.01)	.32
		Weighted mode	0.99 (0.98, 1.00)	.04
Long sleep duration	10	IVW	1.00 (0.99, 1.01)	.33
		Weighted median	1.00 (0.99, 1.01)	.17
		MR-Egger	0.99 (0.98, 1.01)	.33
		Weighted mode	0.99 (0.99, 1.00)	.19
Short sleep duration	10	IVW	0.99 (0.98, 1.00)	.15
		Weighted median	0.99 (0.98, 1.00)	.02
		MR-Egger	0.99 (0.97, 1.02)	.52
		Weighted mode	0.99 (0.98, 1.00)	.04
Insomnia	10	IVW	1.00 (0.99, 1.01)	.99
		Weighted median	0.99 (0.98, 1.01)	.32
		MR-Egger	0.99 (0.98, 1.01)	.56
		Weighted mode	1.00 (0.98, 1.01)	.59

IVW = inverse variance weighted.

**Table 6:** Heterogeneity and pleiotropy test results of sleep characteristics on hyperthyroidism.

Exposure	Method	Heterogeneity		Pleiotropy	
		*Q*	*P*	Intercept	*P*
Time type	MR-Egger	6.73	.67	0.01	.63
	IVW	6.98	.73		
Daytime sleepiness	MR-Egger	11.03	.20	0.02	.39
	IVW	12.19	.20		
Long sleep duration	MR-Egger	4.61	.60	0.02	.56
	IVW	4.99	.66		
Short sleep duration	MR-Egger	3.51	.32	−0.06	.35
	IVW	4.90	.30		
Insomnia	MR-Egger	6.59	.47	−0.01	.88
	IVW	6.62	.58		

IVW = inverse variance weighted.

**Table 7:** Heterogeneity and pleiotropy test results of sleep characteristics on hypothyroidism.

Exposure	Method	Heterogeneity		Pleiotropy	
		*Q*	*P*	Intercept	*P*
Time type	MR-Egger	21.72	.06	−0.03	.05
	IVW	32.15	.07		
Daytime sleepiness	MR-Egger	22.44	.06	0.01	.49
	IVW	23.91	.06		
Long sleep duration	MR-Egger	5.55	.70	0.02	.06
	IVW	10.30	.33		
Short sleep duration	MR-Egger	0.23	.99	−0.03	.23
	IVW	2.22	.82		
Insomnia	MR-Egger	9.28	.32	0.01	.60
	IVW	9.62	.38		

**Table 8:** Heterogeneity and pleiotropy test results of hyperthyroidism on sleep characteristics.

Outcomes	Method	Heterogeneity		Pleiotropy	
		*Q*	*P*	Intercept	*P*
Time type	MR-Egger	10.92	.08	−0.01	.78
	IVW	11.27	.06		
Daytime sleepiness	MR-Egger	6.26	.10	−0.01	.64
	IVW	6.80	.15		
Long sleep duration	MR-Egger	2.64	.45	−0.01	.87
	IVW	2.68	.61		
Short sleep duration	MR-Egger	2.60	.45	0.01	.23
	IVW	4.86	.30		
Insomnia	MR-Egger	2.67	.45	0.01	.34
	IVW	3.96	.41		

**Table 9:** Heterogeneity and pleiotropy test results of hypothyroidism on sleep characteristics.

Outcomes	Method	Heterogeneity		Pleiotropy	
		*Q*	*P*	Intercept	*P*
Time type	MR-Egger	13.76	.09	−2.68e^−^05	.99
	IVW	13.76	.13		
Daytime sleepiness	MR-Egger	18.45	.06	0.01	.82
	IVW	18.57	.07		
Long sleep duration	MR-Egger	10.04	.26	0.01	.55
	IVW	10.53	.31		
Short sleep duration	MR-Egger	33.93	4.18e^−05^	−0.01	.89
	IVW	34.01	8.90e^−05^		
Insomnia	MR-Egger	6.85	.55	0.01	.48
	IVW	7.40	.60		

### 3.2. Short sleep duration and thyroid function

**Figure 2.**
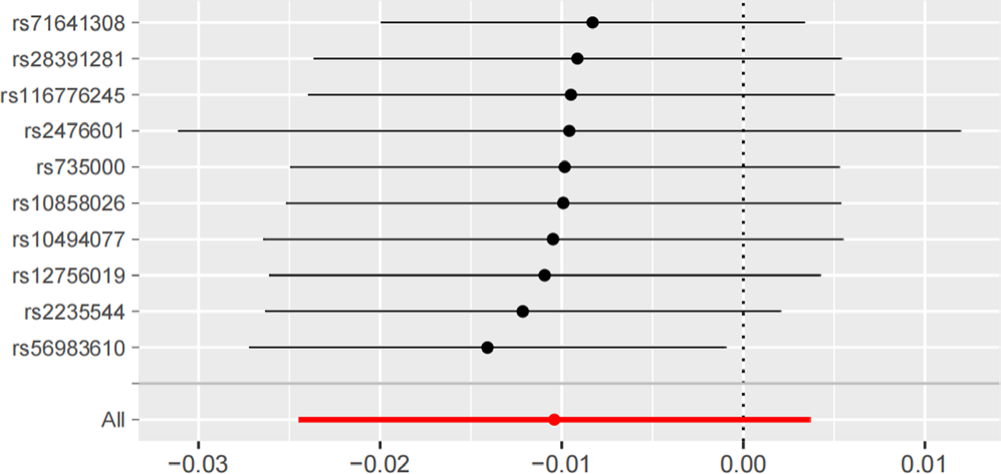
Leave-one-out sensitivity analysis for hypothyroidism on short sleep duration.

The MR results in this section assessed the causal effect of short sleep duration on thyroid function based on 5 short sleep duration-associated SNPs. The IVW results showed that short sleep duration was not associated with the risk of hyperthyroidism (OR = 0.30, 95% CI: 6.15e^−03^–1.46e^+01^, *P* = .54), hypothyroidism (OR = 1.29, 95% CI: 0.35–4.80, *P* = .71) risk was not relevant (Tables 2 and 3), and the other 3 methods were consistent with the IVW results. In the reverse MR study, IVW results showed that hyperthyroidism (OR = 0.99, 95% CI: 0.98–1.00, *P* = .19) and hypothyroidism (OR = 0.99, 95% CI: 0.98–1.00, *P* = .15) were not associated with the risk of short sleep duration (Tables 4 and 5). Whereas, the weighted median method and weighted mode results suggested that hypothyroidism may be associated with the risk of short sleep duration, weighted median method (OR = 0.99, 95% CI: 0.98–1.00, *P* = .02), and weighted mode (OR = 0.99, 95% CI: 0.98–1.00, *P* = .04). In addition, the results of the MR-Egger test for horizontal polytropy indicated that the MR analyses were not affected by any potential effects of horizontal polytropy (*P* > .05; Tables 6–9). Finally, the leave-one-out sensitivity analysis confirmed the robustness of the MR results (Fig. 2).

### 3.3. Long sleep duration and thyroid function

We assessed the causal effect of long sleep duration on thyroid function based on 8 sleep duration-associated SNPs. IVW results showed that long sleep duration was not associated with the risk of hyperthyroidism (OR = 0.55, 95% CI: 8.59e^−03^–35.56, *P* = .78), hypothyroidism (OR = 2.11, 95% CI: 0.41–11.02, *P* = .37; Tables 2 and 3), and the other 3 methods were consistent with the IVW results. In the reverse MR study, IVW results showed that hyperthyroidism (OR = 1.00, 95% CI: 0.99–1.01, *P* = .39), and hypothyroidism (OR = 1.00, 95% CI: 0.99–1.01, *P* = .33) were not associated with the risk of long sleep duration (Tables 4 and 5). The other 3 methods were consistent with IVW results. In addition, the results of the MR-Egger test of horizontal polytropy indicated that the MR analysis was not affected by any potential effect of horizontal polytropy (*P* > .05; Tables 6–9). Finally, the leave-one-out method sensitivity analysis confirmed the robustness of the MR results.

### 3.4. Daytime sleepiness and thyroid function

**Figure 3.**
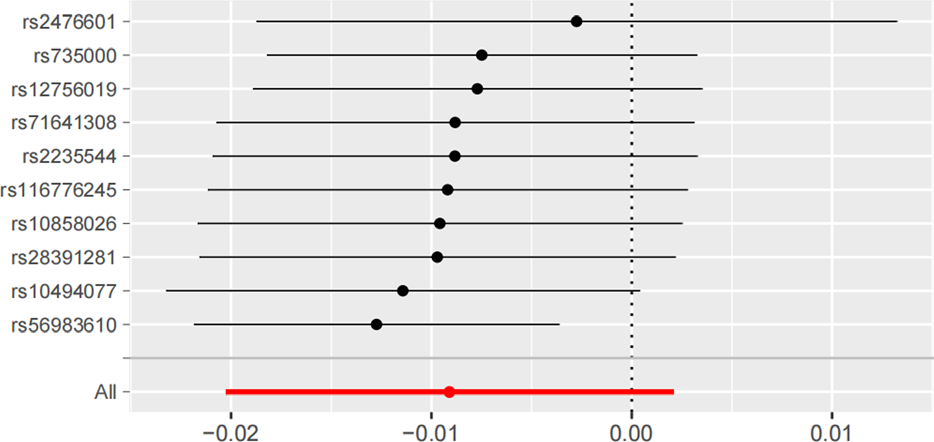
Leave-one-out sensitivity analysis for hypothyroidism on daytime sleepiness.

The causal relationship between daytime sleepiness and thyroid function were assessed by means of 10 SNPs associated with daytime sleepiness. IVW results showed that daytime sleepiness was not associated with the risk of hyperthyroidism (OR = 0.61, 95% CI: 0.05–6.89, *P* = .69), hypothyroidism (OR = 0.90, 95%CI: 0.26–3.11, *P* = .87; Tables 2 and 3), and additional 3 methods were consistent with the IVW results. In the reverse MR study, IVW results showed that hyperthyroidism (OR = 1.01, 95% CI: 0.99–1.02, *P* = .49), hypothyroidism (OR = 0.99, 95% CI: 0.98–1.00, *P* = .11) were not associated with the risk of long sleep duration (Tables 4 and 5). Whereas, the weighted median method and weighted mode results suggested that hypothyroidism may be associated with the risk of daytime sleepiness, weighted median method (OR = 0.99, 95% CI: 0.98–1.00, *P* = .01), and weighted mode (OR = 0.99, 95% CI:0.98–1.00, *P* = .04). In addition, the results of the MR-Egger test for horizontal polytropy indicated that the MR analyses were not affected by any potential effects of horizontal polytropy (*P* > .05; Tables 6–9). Finally, the leave-one-out sensitivity analysis confirmed the robustness of the MR results (Fig. 3).

### 3.5. Insomnia and thyroid function

In examining the effect of insomnia on the risk of hyperthyroidism and hypothyroidism, 10 SNPs remained after a series of exclusions for MR analysis. In the MR assessment, IVW showed that insomnia was associated with hyperthyroidism (OR = 0.53, 95% CI: 1.00e^−01^–2.80, *P* = .46), and hypothyroidism (OR = 0.92, 95% CI: 0.47–1.79, *P* = .80; Tables 2 and 3). In the reverse MR study, hyperthyroidism (OR = 1.01, 95% CI: 0.99–1.02, *P* = .33), and hypothyroidism (OR = 1.00, 95% CI: 0.99–1.01, *P* = .99) were not associated with the risk of insomnia (Tables 4 and 5). No horizontal pleiotropy was detected using the MR-Egger test (*P* > .05; Tables 6–9). Finally, leave-one-out sensitivity analysis confirmed the robustness of the MR results.

## 4. Discussion

We employed a 2-sample MR approach to comprehensively assess the impact of sleep characteristics on thyroid function. The findings from the inverse MR analysis indicated that only the results from the weighted median and weighted mode methods suggested a potential causal link between hypothyroidism and short sleep duration, as well as daytime sleepiness; the remaining results did not show a causal association. Our study did not provide compelling evidence supporting the genetic prediction of a causal relationship between sleep characteristics and thyroid function.

Research investigating the correlation between disrupted sleep patterns and abnormal thyroid function has demonstrated clinical relevance in monitoring thyroid function in individuals with insomnia and assessing the sleep quality of patients with thyroid disorders. Abnormal sleep and abnormal thyroid function are common clinical disease states.^[33]^ Thyroid hormone secretion and conduction are important for human growth and development and affect many different bodily physiological functions to varied degrees.^[34]^ Furthermore, the majority of hormone production follows a 24-hour circadian rhythm, and sleep has varied effects on how this rhythm is regulated.^[35]^ Thyroid stimulating hormone (TSH) levels are notably influenced by sleep, and FT3 has a circadian cycle fluctuation that is compatible with TSH.^[10,36]^ Research has highlighted the intricate relationship between hormones and sleep in human physiological functions, as well as the dynamic interaction between the endocrine system and sleep.^[37,38]^ It is well-established that sleep significantly impacts the release of thyroid hormones, and conversely, thyroid hormone levels play a crucial role in determining the quality of sleep.

According to Sridhar et al,^[39]^ there exists a significant correlation between elevated thyroid hormone levels and delayed sleep onset, shortened sleep duration, and daytime drowsiness. Prolonged sleep latency was notably associated with alterations in mood, bowel movements, and appetite mediated by thyroid hormones. Similarly, individuals experiencing tremors due to heightened thyroid hormone levels reported difficulties in maintaining sleep. In a study by Chattopadhyay et al,^[40]^ a cohort of 36 Indian patients with newly diagnosed Graves’ disease and concurrent psychiatric conditions was evaluated. Among them, 41% had generalized anxiety disorder, 16% had obsessive-compulsive disorder, and 16% had undifferentiated mood disorder. The patients had recently received a diagnosis of Graves’ disease. Common patient complaints included feelings of worry, irritability, and insomnia. The participants were divided into 2 treatment groups for the trial. One group received antipsychotic and antithyroid medications, while the other group received only antithyroid drugs. The study reported improvements in symptoms of insomnia, irritability, and anxiety in both treatment groups. The degree of symptom relief did not, however, differ significantly between the 2 groups. TSH and FT3 levels were found to be directly correlated with the intensity of insomnia symptoms in 1 study.^[41]^ The association between untreated subclinical hypothyroidism and insomnia has been reported in a number of studies. Song et al^[42]^ discovered that people with low TH levels frequently had poorer sleep quality and longer sleep latency than people with normal thyroid function. These results provide credence to the notion that irregular thyroid function is closely associated with sleep disturbances, mental health issues, and other issues.

The present study, on the other hand, does not substantiate the idea that irregular sleep patterns contribute to an increased risk of hyperthyroidism and hypothyroidism. We assessed the results considering additional variables. Initially, self-reporting without stringent rating criteria was employed to assess participants’ sleep durations in studies examining the relationship between thyroid function and sleep. Consequently, there could be disparities between the reported figures and the actual sleep durations of the participants, and the outcomes may vary based on seasonal factors and recall bias.^[43]^ Prior research has established a connection between sleeplessness and mental health conditions like depression and anxiety.^[44]^ In other words, sleeplessness can contribute to the emergence of anxiety and despair. Furthermore, some research has indicated that thyroid malfunction, which is a risk factor for depression, may be brought on by anxiety and depression.^[45,46]^ Thus, the mechanism of link between thyroid problems and insomnia may involve some interaction between both components. This suggests that while sleep may indirectly affect thyroid function through other factors, it is not directly linked to an increased risk of aberrant thyroid function.

Moreover, there is no evidence suggesting a direct link between hyperthyroidism, hypothyroidism, and insomnia. Individuals with thyroid dysfunction may not necessarily experience poor sleep quality solely due to their condition, but rather due to a combination of factors. Patients with thyroid disorders often encounter feelings of anxiety and depression, which are commonly associated with insomnia at a higher frequency than in the general population.^[47]^ Therefore, conditions that can disrupt sleep, such as anxiety, depression, or other medical conditions, may be triggered or exacerbated by hyperthyroidism or hypothyroidism. Additionally, a well-documented instance of thyroid dysfunction contributing to sleep disturbances is the heightened susceptibility to restless legs syndrome resulting from irregular thyroid activity. Individuals with restless legs syndrome experience discomfort or unpleasant sensations in their legs or body when at rest, leading to symptoms that typically emerge during sleep and can lead to insomnia and sleep disruptions.^[48]^ Furthermore, the fact that symptoms of thyroid hormone insufficiency can cause sleeplessness is 1 reason hypothyroidism and sleep disturbances may frequently co-occur. For instance, hypothyroidism is linked to symptoms like increased anxiety, cold intolerance, and soreness in the muscles and joints that might prevent you from getting enough sleep. Budhiraja et al^[49]^ suggest that the greater the number of comorbidities, the higher the risk of comorbidity. Even if thyroid hormone deficiency does not directly contribute to insomnia, the various symptoms associated with thyroid dysfunction can easily exacerbate sleep difficulties and reduce a person’s ability to obtain quality sleep.

It is important to note that the weighted mode and weighted median approaches in our reverse magnetic resonance imaging investigation suggested a potential causal link between hypothyroidism and daytime sleepiness and short-duration sleep. Furthermore, as is well known, the primary evaluation criterion for this investigation is the IVW result; a favorable outcome is possible if 50% of the SNPs in the weighted median and weighted mode approaches satisfy the necessary final criteria. Furthermore, the Akatsu et al^[50]^ study did not find any association between subclinical hypothyroidism and sleep quality. Thus far, no research has been able to thoroughly examine or pinpoint the precise mechanism via which lower thyroid hormone levels could impact the quality of sleep. When these factors are considered and scientific evidence is considered, all we can conclude is that hypothyroidism and brief sleep intervals and daytime sleepiness may be related in a way that warrants further investigation.

The present study boasts several strengths. Firstly, it is the first known study to utilize MR for the analysis of sleep characteristics and thyroid function. Furthermore, it conducted a bidirectional causality study, significantly enhancing the existing body of research in this field by exploring the causal relationship between sleep and thyroid function. Secondly, in comparison to previous observational studies on sleep and thyroid function, the study’s MR design was less susceptible to confounding variables. Thirdly, the IVs utilized in this study was statistically robust, derived from a large sample size GWAS database, enabling accurate estimation of causality.

However, the study is not without limitations. Firstly, it was unable to investigate potential variations between different races and nations, as the subjects in the GWAS dataset were of European descent. Secondly, despite the absence of pleiotropy in our sensitivity analysis, the potential for multiple effects in MR studies was not entirely ruled out. Additionally, the study did not delve into the molecular mechanisms underlying the relationship between thyroid function and sleep. Therefore, future research necessitates additional genetic data and large-scale investigations to elucidate the intricate connection between thyroid function and sleep.

## 5. Conclusion

This bidirectional MR indicates that hypothyroidism should not be attributed to changes in sleep type, but there is a potential causal relationship between short sleep duration, daytime sleepiness, and hypothyroidism. This helps to better understand the relationship between sleep and thyroid function, as well as potential evidence for clinical interventions in hypothyroidism, which requires further investigation to elucidate its nature and the underlying mechanisms of this discovery.

## Acknowledgments

The authors would like to thank the UK Biobank Sleep Traits and the Finnish Genetic Alliance for generously sharing GWAS summary statistics. We thank the Natural Science Foundation of Shandong Province, the special funding for Mount Tai Scholar Project, and the Shandong Medical & Health Science and Technology Development Foundation for financial support.

## Author contributions

**Conceptualization:** Zonghang Jia.

**Data curation:** Zonghang Jia.

**Formal analysis:** Zonghang Jia.

**Funding acquisition:** Yujie Li.

**Investigation:** Yujie Li.

**Methodology:** Zonghang Jia, Zhonghui Li.

**Software:** Zonghang Jia, Zhonghui Li.

**Supervision:** Yujie Li.

**Validation:** Zhonghui Li, Yujie Li.

**Visualization:** Zonghang Jia.

**Writing – original draft:** Zonghang Jia.

**Writing – review & editing:** Zonghang Jia, Zhonghui Li.
